# Geographic Variation in *Festuca rubra* L. Ploidy Levels and Systemic Fungal Endophyte Frequencies

**DOI:** 10.1371/journal.pone.0166264

**Published:** 2016-11-15

**Authors:** Serdar Dirihan, Marjo Helander, Henry Väre, Pedro E. Gundel, Lucas A. Garibaldi, J. Gonzalo N. Irisarri, Irma Saloniemi, Kari Saikkonen

**Affiliations:** 1 Department of Biology, University of Turku, Turku, Finland; 2 Natural Resources Institute Finland (Luke), Turku, Finland; 3 Botanical Museum, Finnish Museum of Natural History, University of Helsinki, Helsinki, Finland; 4 IFEVA, Facultad de Agronomía, Universidad de Buenos Aires, CONICET, Buenos Aires, Argentina; 5 Grupo de Investigación en Agroecología (AGRECO), Sede Andina, Universidad Nacional de Río Negro (UNRN) and Consejo Nacional de Investigaciones Científicas y Técnicas (CONICET), San Carlos de Bariloche, Río Negro, Argentina; National Cheng Kung University, TAIWAN

## Abstract

Polyploidy and symbiotic *Epichloë* fungal endophytes are common and heritable characteristics that can facilitate environmental range expansion in grasses. Here we examined geographic patterns of polyploidy and the frequency of fungal endophyte colonized plants in 29 *Festuca rubra* L. populations from eight geographic sites across latitudes from Spain to northernmost Finland and Greenland. Ploidy seemed to be positively and negatively correlated with latitude and productivity, respectively. However, the correlations were nonlinear; 84% of the plants were hexaploids (2n = 6x = 42), and the positive correlation between ploidy level and latitude is the result of only four populations skewing the data. In the southernmost end of the gradient 86% of the plants were tetraploids (2n = 4x = 28), whereas in the northernmost end of the gradient one population had only octoploid plants (2n = 8x = 56). Endophytes were detected in 22 out of the 29 populations. Endophyte frequencies varied among geographic sites, and populations and habitats within geographic sites irrespective of ploidy, latitude or productivity. The highest overall endophyte frequencies were found in the southernmost end of the gradient, Spain, where 69% of plants harbored endophytes. In northern Finland, endophytes were detected in 30% of grasses but endophyte frequencies varied among populations from 0% to 75%, being higher in meadows compared to riverbanks. The endophytes were detected in 36%, 30% and 27% of the plants in Faroe Islands, Iceland and Switzerland, respectively. Practically all examined plants collected from southern Finland and Greenland were endophyte-free, whereas in other geographic sites endophyte frequencies were highly variable among populations. Common to all populations with high endophyte frequencies is heavy vertebrate grazing. We propose that the detected endophyte frequencies and ploidy levels mirror past distribution history of *F*. *rubra* after the last glaciation period, and local adaptations to past or prevailing selection forces such as vertebrate grazing.

## Introduction

Biogeographic generalizations on the factors responsible for patterns of species’ ranges are largely based on comparisons of closely related species [1]. For example, polyploidy appears to be positively associated with latitude, altitude and recent deglaciations [2–5], and biotic interactions have been connected to adaptive radiation of plants [6–8]. Although different ploidy levels have commonly been documented within species as well, extensive studies on geographic species-specific ploidy distributions and importance of biotic interactions are mostly lacking [9], and sporadic findings are often conflicting.

Grasses are a perfect model for studies on geographic ploidy distributions and importance of biotic interactions because they cover higher area of land than any other group of plants across all the continents except Antarctica [10]. We selected red fescue (*Festuca rubra* L. *sensu lato*) as a model species for our study. First, it is a wild perennial Eurasian grass widely distributed and phenotypically variable in the Northern hemisphere. Plants falling into morphologically distinguishable categories are often inconsistently classified as both species and subspecies showing local adaptations [11, 12]. Available literature suggests extensive hybridization between (sub)species, potentially resulting in observed natural polyploids (2n = 14, 21, 28, 42, 49, 56, 64 and 70) [11,12]. Interfertile plants exhibit striking morphological variation and part of the ecotypic diversity is suggested to be related to the polyploidization [13]. Second, red fescue is well known for its variable and occasionally high frequencies of systemic fungal endophytes [14–17]–plant associated fungi that are suggested to act as defensive plant mutualists and thereby expand distribution range of the host grass [7, 18–21].

Both polyploidy and systemic, vertically in germline transmitted *Epichloë* endophytes are common grass characteristics that can be adaptive to various environmental conditions [2, 4, 6, 7, 22–24]. Polyploidy, the multiplication of the complete set of chromosomes, can bestow adaptive potential and evolutionary flexibility on plants and thereby improve their competitive and invasive capacity into northern latitudes [4, 23, 25]. Possessing more than two sets of chromosomes can cause heterosis, shield polyploids from deleterious effects of mutations for example by reducing the incidence of homozygous recessives, and buffer against inbreeding depression and genetic drift [2, 5, 23]. Because polyploids are usually unable to interbreed with their diploid conspecifics, polyploidy is recognized as one of the major mechanisms of sympatric speciation [5] and in some species the number of chromosomes appears to be positively correlated with latitude or altitude [2–4, 26]. Recently, the role of polyploidization as a modulator of adaptive symbiosis between plants and microbes has been recognized [27]. For example, polyploidization can affect biotic interactions through changes in the chemical profile of the plant [8]. However, the empirical evidence is variable and partly contradictory [28–30], and the question how ploidy-driven adaptations to environmental conditions and microbial interactions codetermine plant distribution is unknown.

Similar to polyploidy, *Epichloë* endophytes examined in this study can also drive the geographic distribution of host grasses [31]. Grass endophytes of the genus *Epichloë* are common symbionts of cultivated and wild Pooideae grass species. In the symbiosis endophytic fungus grows systemically and asymptomatically throughout the aboveground plant parts, and depending on the species it may be transmitted either vertically via host seeds and/or horizontally by sexual spores [6, 7]. In many species, the fungus is mostly asexual and is transmitted vertically from mother plant to its offspring. Vertical transmission is supposed to promote fidelity between partners and lead to mutualistic symbiosis because the fitness of the heritable fungus and the host grass is tightly linked [32–38]. Thus, the symbioses are commonly thought to be mutualistic. Numerous studies have demonstrated that *Epichloë* species can increase grass resilience to drought, flooding, pathogens and herbivores, and thus promote their competitive ability in grass communities [14, 18, 21, 39–41]. The defensive mutualism against herbivores due to mycotoxins appears to provide the most prevalent ground for mutualistic endophyte-grass interactions [6, 7, 20, 21, 42, 43]. However, an increasing number of empirical studies has revealed that the symbiosis can range from antagonistic to mutualistic interactions, and mutualism is less frequent in wild grasses compared to agronomic grasses in nutrient-rich environments [20, 21, 38, 44, 45].

This study aims to reveal potential linkages between geographic patterns of polyploidy and plant-fungal endophyte symbiosis. We explore both ploidy levels and endophyte frequencies in red fescues over a wide range of environments and latitudes across Europe. The benefits from *Epichloë* species are found to be positively correlated with high nutrient availability and productivity [20, 46], and polyploidy is believed to increase towards less productive higher latitudes and altitudes [4, 23]. Thus, we studied primary productivity (the normalized difference vegetation index, NDVI) of our study sites [47]. We also hypothesize that ploidy levels and the frequencies of endophyte symbiosis would be correlated, because polyploidization can modulate plant-microbe interactions and both polyploidization and systemic grass endophytes can promote host fitness. Because benefits of endophytes are particularly obvious in high nutrient environments, we may assume that the positive correlation should be stronger in environments with the highest primary production. This assumption is supported by a recent broad-scale study [46] suggesting that primary production is positively associated with the occurrence of systemic grass endophyte symbiosis.

## Materials and Methods

More than one thousand red fescue (*Festuca rubra* L.) plants in total were collected from 29 populations (10–70 plants/population) from eight geographic sites across Europe (Spain, Switzerland, southern Finland, Faroe Islands, Iceland, two areas in northern Finland and Greenland; Table 1). Plants were not collected from national parks or other protected areas requiring permissions. In Nordic countries “everyman’s right” gives everyone rights to access, enjoy for recreation and collect unprotected plants, berries and mushrooms in a way which does not damage the environment or disturb others regardless who owns or occupies the land. Plants from Spain and Switzerland were collected from public land. Geographical coordinates, altitude from the sea level and features of the site were recorded for each population (Table 1).

**Table 1:** The collection sites of *Festuca rubra* plants and their attributes. N = number of collected plants. Mean NDVI = mean normalized difference vegetation index estimated from year 2000 to 2012. Inf. % = percentage of endophyte infected plants (endophyte frequency) in population. 4x %, 6x % and 8x % = percentage of tetraploids, hexaploids and octoploids, respectively, in population.

Population code	Geographic site	Population	N	Geographic coordinates	Altitude (m a.s.l.)	Mean NDVI	Inf. %	4x %	6x %	8x %	Features of the site	Grazing
SP1	Spain	Cáceres	31	N 40°12'1'' W 5°45'11''	768	0,59	81	100	0	0	Xerophytic forest	High (cattle)
SP2	Spain	Salamanca 1	27	N 40°56'20'' W 6°7'6''	863	0,57	67	78	22	0	Meadow	High (cattle)
SP3	Spain	Salamanca 2	37	N 40°58'24'' W 5°57'33''	812	0,51	59	81	3	16	Meadow	High (cattle)
SW1	Switzerland	Andermatt	26	N 46°32'19'' E 8°40'31''	1500	0,42	23	0	96	4	Meadow with natural grassland vegetation, slope facing east	High (cattle)
SW2	Switzerland	Biez	25	N 46°37'42'' E 8°35'26''	1600	0,41	36	0	100	0	Meadow with natural grassland vegetation, slope facing north-west	High (cattle)
SW3	Switzerland	Piasca	23	N 46°53'56'' E 8°42'9''	1850	0,19	22	0	100	0	Meadow with natural grassland vegetation, sawed every second year, slope facing south	High (cattle)
FI1	Southern Finland	Hanko 1	42	N 59°50'23'' E 23°13'40''	0	0,55	0	2	98	0	Meadow along the coast	Low
FI2	Southern Finland	Hanko 2	44	N 59°50'27'' E 23°13'15''	0	0,53	0	0	93	7	Meadow along the coast	Low
FI3	Southern Finland	Hanko 3	40	N 59°53'0'' E 23°5'52''	0	0,43	0	5	95	0	Meadow along the coast	Low
FO1	Faroe	Sandoy	39	N 61°50'11'' W 6°51'21''	69	0,42	21	3	97	0	Meadow	High (sheep)
FO2	Faroe	Nolsoy	41	N 62°1'15'' W 6°41'8''	55	0,33	5	0	98	2	Meadow	High (sheep)
FO3	Faroe	Mykines	37	N 62°5'51'' W 7°40'56''	125	0,16	68	0	100	0	Meadow	High (sheep)
FO4	Faroe	Vagar	24	N 62°6'59'' W 7°26'43''	246	0,30	25	0	83	17	Meadow	High (sheep)
FO5	Faroe	Eysturoy	39	N 62°17'24'' W 7°2'10''	316	0,34	54	5	87	8	Meadow	High (sheep)
FO6	Faroe	Vidoy	32	N 62°22'3'' W 6°32'32''	148	0,31	44	9	91	0	Meadow	High (sheep)
IC1	Iceland	Iceland 1	44	N 64°47'34'' W 21°32'0''	390	0,28	32	2	98	0	Meadow	High (sheep)
IC2	Iceland	Iceland 2	34	N 64°48'52'' W 23°23'14''	10	0,34	32	0	97	3	Meadow	High (sheep)
IC3	Iceland	Iceland 3	42	N 66°1'21'' W 20°23'39''	38	0,30	26	5	95	2	Meadow	High (sheep)
GR1	Greenland	Greenland 1	70	N 69°14'59'' W 53°31'15''	0	0,16	3	0	100	0	Meadow along the coast	Low
GR2	Greenland	Greenland 2	10	N 69°15'27'' W 53°32'40''	0	0,19	0	0	100	0	Meadow along the coast	Low
FI4	Northern Finland 1	Halti 1	22	N 69°15'0'' E 21°24'36''	860	0,20	0	0	100	0	Meadows along rivulets above tree-line with patchy grass and sedge dominated vegetation	Moderate (reindeer)
FI5	Northern Finland 1	Halti 2	42	N 69°15'0'' E 21°19'12''	900	0,11	0	0	0	100		Moderate (reindeer)
FI6	Northern Finland 1	Halti 3	32	N 69°16'12'' E 21°19'12''	920	0,28	0	0	100	0		Moderate (reindeer)
FI7	Northern Finland 2	Kevo 1	34	N 69°38'6'' E 27°5'1''	91	0,31	56	3	97	0	Meadow	High (reindeer)
FI8	Northern Finland 2	Kevo 2	40	N 69°43'56'' E 27°12'0''	85	0,31	75	3	85	13	Meadow	High (reindeer)
FI9	Northern Finland 2	Kevo 3	34	N 69°45'32'' E 26°59'19''	107	0,31	50	0	94	6	Meadow	High (reindeer)
FI10	Northern Finland 2	Kevo 4	42	N 69°54'36'' E 27°1'48''	73	0,27	45	2	98	0	Riverbank	High (reindeer)
FI11	Northern Finland 2	Kevo 5	31	N 69°56'11'' E 26°27'45''	106	0,28	23	0	94	6	Riverbank	High (reindeer)
FI12	Northern Finland 2	Kevo 6	35	N 69°56'41'' E 26°43'22''	85	0,29	20	0	100	0	Riverbank	High (reindeer)

To ensure the proper species identification of the plants and that collected plants represent individual genets, only flowering individuals growing at least 10 meters apart from each other were collected. Plants were dug up with a soil core and placed into plastic bags for transportation. All the collected grasses were planted in 250 ml pots with added peat and sand mix around the original soil core and kept in a greenhouse in Turku University Botanical Garden (60°26’N, 22°10’E) in ambient daylight and 20–24°C (summer time) and 4–8°C (winter time) temperatures.

The collection sites represent a broad biogeographical region varying in terms of latitudes, altitudes, climatic zones (continental, oceanic), biological selection pressures such as grazing (Table 1) and seasonal changes in abiotic environmental conditions. For example, sites in Spain represent grassland and xerophytic forest, both in Mediterranean climate characterized by summer droughts and rainy winters. Sites located on higher latitudes are characterized by stronger seasonal changes in day length and associated light quality limiting primary production [48], short growing seasons in summer and long and cold winters. However, oceanic sites (Iceland and Faroe Islands) strongly affected by the Gulf-stream are characterized by high precipitation year-round, cool summers and relatively mild winters compared to the other sites on the same latitudes.

### Ploidy determination

Ploidy levels of the plants were determined by flow cytometry (FCM) [49]. We used known chromosome counts of *F*. *rubra* plants as references for the FCM results [49]. Plants from different populations (one to three plants for each population) were randomly chosen to microscopically determine cytotype. These reference plants were grown hydroponically to produce fresh root tips. Aseptically cut root tips were pretreated with 1% 1-alphabromonaphtalene and stained in 2% acetic orcein solution [50]. The root tips were then squashed in a drop of 45% acetic acid on the slides and analyzed under microscope. Preparations were mounted with enthalan after the metaphases were photographed.

We sampled a ca. 0.5 cm^2^ leaf piece from each plant. The sample was chopped in a glass Petri dish with an aseptic razor blade in 1 ml ice-cold nuclei isolation buffer (LBO1 in one-step procedure). The suspension was mixed by sucking and discharging with the pipette several times and then filtered into an Eppendorf tube using a 50 μm nylon mesh. DNA fluorochrome stock solution with 50 μl ml^-1^ propidium iodide (PI) and 50 μl ml^-1^ ribonuclease (RNase) was added and incubated on ice one hour before FCM analysis [51]. The 96 well plate-based FCM procedure [49] was carried out using LSR II (Bechton Dickinson San Jose, USA) flow cytometer at the Turku Centre for Biotechnology, Finland. *Pisum sativum* L. ‘Ctirad’ (2C DNA value = 9.09 pg) plants obtained from the Institute of Experimental Botany (Laboratory of Molecular Cytogenetics and Cytometry, Olomouc, Czech Republic), were used as an external reference to determine DNA quantity in pictograms (pg). In addition, known tetraploid and hexaploid *F*.*rubra* plants were used for each FCM run. The FCM channel was determined as G_1_ peak for each sample and DNA ploidy levels were estimated as follows:

$$Ploidy\;level\;of\;sample=\frac{G_{1}\;peak\;flourescence\;of\;sample\;(median)\times G_{1}\;peak\;flourescence\;of\;reference\;(median)}{Ploidy\;level\;of\;reference}$$

The flow cytometric data were measured with Flowing Software version 2.4.1 (Perttu Terho, Turku Centre for Biotechnology, Finland; www.flowingsoftware.com).

### Endophyte detection

Fungal endophyte status (endophyte infected E+ / endophyte free E-) of each of the study plants was detected by plating three leaf sheaths ˗ surface- sterilized by incubation for 1 min in 90% ethanol, 4 min in 4% sodium hypochlorite and 30 s in 90% ethanol ˗ cut into 5 pieces and placed on potato dextrose agar (5% PDA). The Petri dishes were monitored up to three weeks for their systemic endophyte fungal growth. When typical white *Epichloë* fungal endophyte mycelia grew out from several leaf pieces, the plant was considered as endophyte infected [16]. The infection status of individual plants was verified later by staining and microscopic examination of several seeds of each plant. Systemic and vertically via host grass transmitted endophytic fungi are host species-specific. Similarly to other studies, we have identified the fungus associated with red fescue as *E*. *festucae* by comparing the rDNA sequences with Blast searches of GeneBank in our previous studies [16].

### NDVI

**Fig 1:**
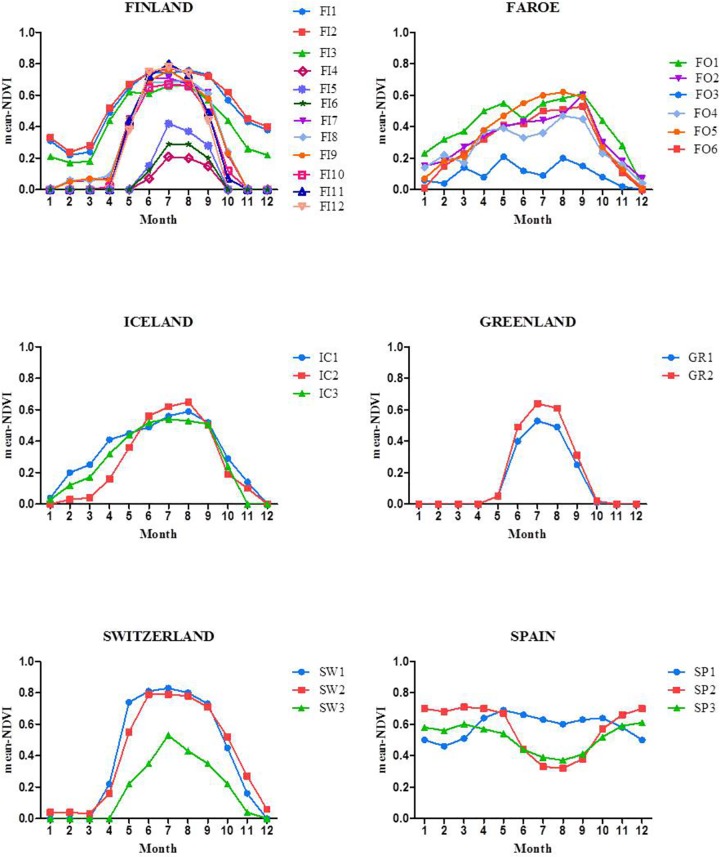
Monthly mean normalized difference vegetation index (mean NDVI) values for the *Festuca rubra* collection sites (for population codes see Table 1) from February 2000 to December 2012.

To compare vegetation productivity of the study sites quantitatively, we calculated the normalized difference vegetation index (NDVI) for each population separately using NASA MODIS satellite images (Fig 1, Table 1). The NDVI accurately estimates functional attributes of the ecosystem such as aboveground net primary production (ANPP), its inter-annual variation and vegetation phenology [47, 52, 53]. NDVI is closely and positively correlated with leaf area and the fraction of photosynthetically active radiation absorbed by green vegetation [54–56]. NDVI data uniquely allows site characterization because it represents the specific consequences of environmental and human effects on vegetation functioning.

We obtained NDVI values from the MODIS project through the MODIS global subsets tool (http://daac.ornl.gov/cgibin/MODIS/GLBVZ1Glb/modissubsetorderglobalcol5.pl). We used the MOD 13 Vegetation Indices product, gridded, 16-day composite images with 250-m pixel size. We extracted two essential attributes of aboveground primary production dynamics by calculating the NDVI average annual integral and its inter-annual coefficient of variation from February 2000 to December 2012 (Fig 1). These traits are known to capture important features of ecosystem functioning [57].

### Statistical analysis

We used linear models (linear regression) to analyse the effects of mean NDVI and latitude on endophyte infection frequencies and mean ploidy of populations. Normality of residuals was checked graphically and using Kolmogorov-Smirnov test results for the models.

We used a logistic regression model to estimate ploidy level, altitude (Alt), latitude (Lat) and their pair-wise interactions as fixed effects on endophyte status (two levels: E+ and E-) using binomial error distribution (logit link). We also included population as a random effect (random intercept model) to account for the fact that individual plants were spatially nested within populations (29 populations in total). We used AIC (Akaike Information Criterion) to select best-fitting models for all combinations of fixed-effect variables. AIC values were obtained based on maximum-likelihood estimates of regression coefficients, because models differed in their fixed structure but shared the same random structure (random intercepts), whereas parameter estimates for final models presented in figures were obtained using the restricted maximum likelihood method [58]. Models were estimated using lmer function of the lme4 package [59] in the R software [60], and AIC and Akaike’s weight for each model of all possible models based on different combinations of the predictor variables were obtained with the dredge function of the MuMln package [61] in the R software.

## Results

**Fig 2:**
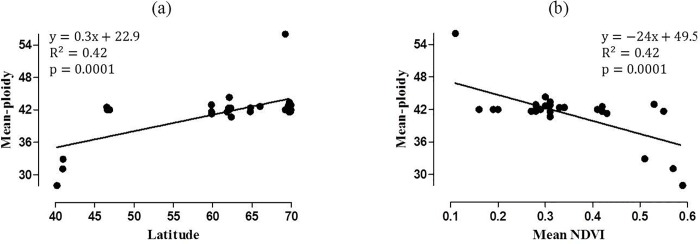
The effect of latitude (a) and mean normalized difference vegetation index (mean NDVI) (b) on mean ploidy of *Festuca rubra* populations. Fitted regression line in (a) is for illustrative purpose since statistical tests are suggestive due to problems of normality in the data.

Overall 84%, 9% and 7% of the plants were hexaploids (2n = 6x = 42), tetraploids (2n = 4x = 28) and octoploids (2n = 8x = 56), respectively. More than one ploidy level was detected in 19 out of 29 populations (Table 1). Ploidy level seems to be partly linked with productivity and latitude; ploidy positively and negatively associated with latitude (p<0.0001) and productivity (p<0.0001), respectively (Fig 2). It is noteworthy, however, that majority of the plants were hexaploids (2n = 6x = 42), and the positive correlation between ploidy level and latitude was not linear, and the correlation is due to the influence of four deviant populations. In the southernmost geographic site, Spain, 86% of the plants were tetraploids (2n = 4x = 28), and in northernmost Finland all of the plants in one population at high altitude (in Halti, ≈ 900m) were octoploids (2n = 8x = 56) (Table 1, Fig 2). Outside these extremes of the latitudinal range, hexaploid plants were dominant. In Greenland, all the plants were hexaploids, and in Switzerland 98% of the plants were hexaploids and only 2% octoploids (Table 1). In the other populations tetraploids and octoploids were sporadically distributed (Table 1).

**Fig 3:**
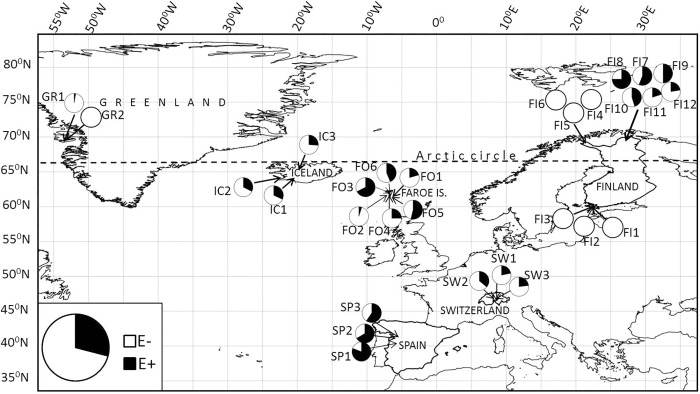
Collection sites and endophyte infection frequencies of *Festuca rubra* populations. Infection frequency circles are shown with the population codes (Table 1).

Endophyte infections were detected in 22 out of 29 red fescue populations (Fig 3). The seven totally endophyte-free populations were the three populations from Hanko in southern Finland, the three populations from Halti in northern Finland and one population from Greenland. Furthermore, only one infected grass was found in the other population in Greenland (Table 1, Fig 3). Overall infection frequency of the study area was 29% but frequencies varied irrespective of ploidy (p = 0.14) (Table 2) among geographic sites, populations within geographic sites and among habitats (Fig 3, Table 1).

**Table 2:** Effects of ploidy, latitude, altitude and interaction between latitude and altitude (Lat x Alt) on endophyte status of *Festuca rubra* plants.

	Estimate	Std. Error	z value	Pr (> |z|)
Intercept	-4.78	5.08	0.94	0.35
Ploidy	0.03	0.02	1.48	0.14
Latitude	0.04	0.08	0.44	0.65
Altitude	0.01	0.00	2.03	0.04*
Lat x Alt	-0.00	0.00	-2.14	0.03*

*, p<0.05

**, p<0.01

***, p<0.001

The highest overall infection frequencies were found in Spain (69%) where occurrence of infections was high in all populations (Fig 3). At Kevo in northern Finland, on average 45% of grasses were infected but infection frequencies were much higher in meadows (60%) compared to nearby riverbanks (29%). The occurrence of infections in Faroe Islands, Iceland and Switzerland were 36%, 30% and 27%, respectively. In these geographic sites variation in endophyte occurrence among populations was considerable only in Faroe Islands varying from 5% to 68% (Table 1, Fig 3).

**Fig 4:**
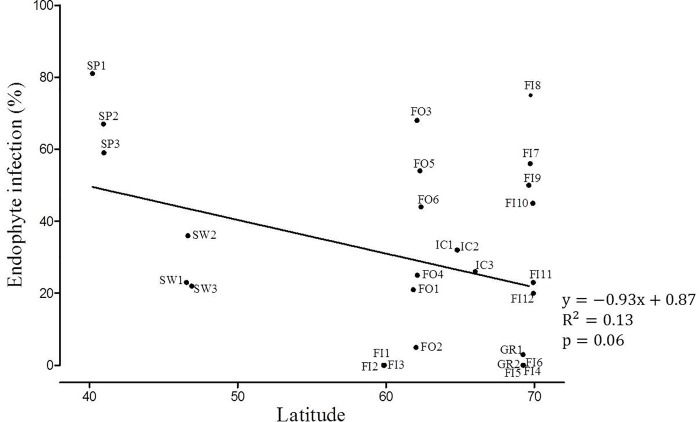
The relationship between latitude and endophyte infection frequencies of *Festuca rubra* populations. Dots are shown with the population codes (Table 1). Fitted regression line is shown, but statistical test is suggestive due to problems of normality in the data.

Latitude appears not to be linked to the detected variation in endophyte occurrence (p = 0.65) (Table 2, Fig 4). Instead, infection frequencies appear to be associated with altitude (p = 0.04) but interactively with latitude (p = 0.03) (Table 2). The sampling was not, however, designed to test the importance of altitude and thus, these results remain inconclusive. In populations collected from low latitudes, in Spain and Switzerland, the endophyte frequencies were lowest in Switzerland where all collected populations were from high altitude (Table 1). In contrast, elsewhere infection frequencies varied irrespective of altitude. For example, populations collected from Greenland and Hanko, both situated at sea level, and Halti situated at ≈900 m above sea level were endophyte-free (Table 1) suggesting the altitude cannot account for patterns of endophyte occurrence.

**Fig 5:**
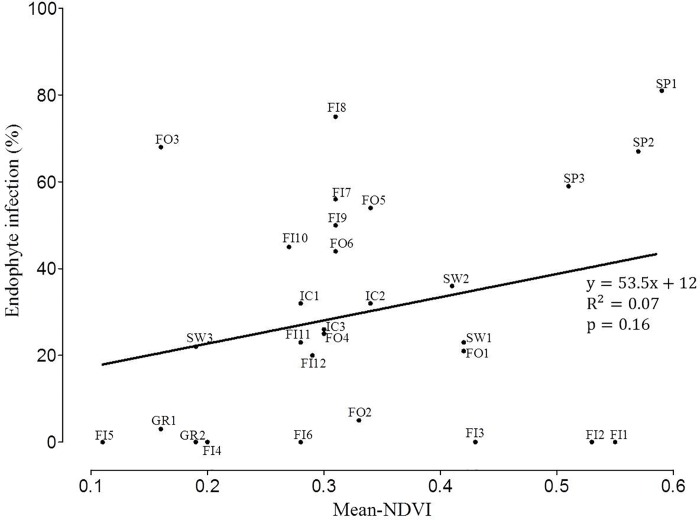
Regression plot of mean normalized difference vegetation index (mean NDVI) against endophyte infection frequencies of *Festuca rubra* populations. Dots are shown with the population codes (Table 1).

**Fig 6:**
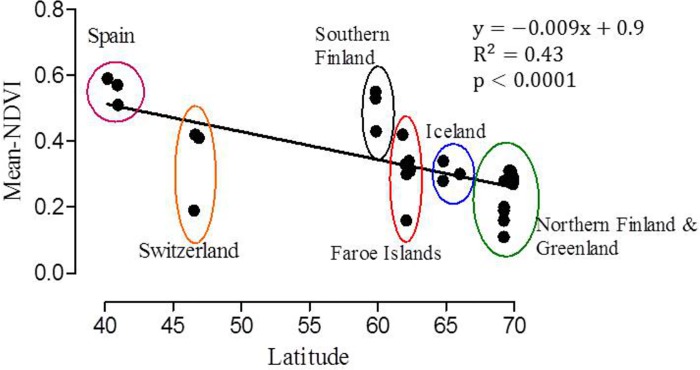
Regression plot of latitude against mean normalized difference vegetation index (mean NDVI) of *Festuca rubra* populations circled by geographic sites (Table 1).

Endophyte frequencies of the grass populations were not associated with productivity (p = 0.16) (Fig 5). Normalized difference vegetation index (NDVI) values varied among geographic sites rather than along latitude (Table 1, Fig 6). The highest geographic site specific mean-NDVI values 0.51, 0.56 and 0.31 were estimated for southern Finland, Spain and Iceland-Faroe Islands, respectively (Table 1). Corresponding endophyte infection frequencies were 0% (southern Finland), 69% (Spain), 30% (Iceland) and 36% (Faroe Islands) demonstrating that overall productivity of the geographic site is unlikely to be linked with endophyte infection frequencies in red fescue populations. Monthly mean NDVI estimates, however, clearly demonstrate that primary production is seasonally limited in all the other study sites except in Spain (Fig 1).

## Discussion

Our results do not support the hypotheses that polyploidization and the occurrence of systemic fungal endophytes in red fescue show latitudinal gradients, or that they are correlated with each other particularly in environments with high primary production. Although ploidy positively correlated with latitude (p<0.0001) as suggested by the literature [2–4], the positive correlation is due to influence of four out of 29 populations located in eight geographic sites (Fig 2). Consistently with our hypothesis, tetraploid plants (2n = 4x = 28) dominated southernmost populations in Spain and octoploids (2n = 8x = 56) one of the northernmost population situated in high altitude in Halti in Finland. However, hexaploids (2n = 6x = 42) predominated all the other geographic sites, and tetraploids and octoploids were sporadically distributed across the latitudes.

Contrary to the prediction that the occurrence of systemic fungal endophytes in red fescue should show a latitudinal gradient and correlate with polyploidization, the frequencies of endophyte infected grasses were highly variable among populations (Table 1, Fig 3). All examined plants collected from southern Finland and from Halti in northern Finland were endophyte-free, and only one plant collected from Greenland hosted endophyte whereas the highest infection frequencies were detected in the southern and northernmost populations of the study area, in Spain and in Kevo in northern Finland. In addition, contrary to the prediction that polyploidization and endophyte infection frequencies should correlate with each other particularly in environments with high primary production, the variation in productivity estimates (NDVI) was not strongly linked with detected infection frequencies (p = 0.16) (Fig 5). In these wild grass populations resource availability appears not to be the primary driving force promoting polyploidization or grass-endophyte symbiosis.

We propose that the discrepancy between studies examining drivers of *Epichloë* endophyte symbioses success is partly caused by scale and sampling differences among studies. For example, in a recent study [46] pooling 1008 records from 48 cool season grass species and their population specific endophyte frequencies from a broad latitudinal gradient, primary productivity was found to be positively correlated with fungal endophyte occurrence in grasses globally. Accordingly, the prevalence of endophyte symbiosis was negatively correlated with latitude because primary production was negatively correlated with latitude [46]. As studies of endophyte occurrence in different grass species along latitudinal gradients accumulate, however, the effects of latitude appear to be variable [14–16]. Both grazing and altitude have been shown to differently affect the distribution patterns of three fescue species (*Festuca rubra*, *F*. *ovina* and *F*. *vivipara*) in Sweden [15]. Altitude and grazing negatively and positively correlated with endophyte frequency of *F*. *rubra*, respectively. In contrast, endophyte occurrence positively correlated with altitude in *F*. *ovina* populations. Neither grazing nor altitude appeared to play role in endophyte symbiosis with *F*. *vivipara* but the number of sample sites was insufficient for statistical analyses of the data. In Finland, the occurrence of *Epichloë* endophytes in *F*. *rubra* and *F*. *ovina* is found to be highest in subarctic areas at the northernmost end of the latitudinal gradient of the studies [14, 16]. For example, in an intensive survey [16] including 86 study sites (in total 2514 plants) across Finland endophytes were detected only occasionally in hemiboreal and boreal areas whilst populations with high endophyte frequencies were found in subarctic Finland. In addition, the prevalence of endophytes varied locally between habitats being highest in meadows. These results call importance for large-scale studies taking into account adaptive radiation of the species and the fact that selection can operate on the endophyte and the host grass individually or in concert as a phenotypic unit of symbiotum.

We propose that one explanation for the detected ploidy and infection patterns in this study is the distribution history of the plants during the 10 000 years since the last glaciation period. A critical difference between these two heritable plant traits, in terms of their evolution, is that endophyte cannot be gained without the presence of infection source whereas polyploidization can naturally arise in plant lineage. Accordingly, we may assume that the window for endophyte infections is ultimately determined by infections in glacial refugia and/or the distribution of endophyte infected plants into the examined geographic sites. Thus, the original infection status of the founder population largely determines whether ploidy levels and endophyte frequencies can co-evolve and correlate with each other in ecological time. For example, populations collected from Greenland and Halti in northern Finland, where fescues are known to reproduce mainly by vegetative propagules [16] can consist of few clones representing small endophyte-free founder populations. Such founder effects can have fundamental effects on adaptive radiation of grasses because the grass genets can survive and drive the genetic population structure of grasses over hundreds of years [62].

Our results reflect how the founder populations have been tested by past and prevailing selection forces such as edaphic factors and biotic interactions. We propose that endophyte infection frequencies represent adaptation to environmental conditions. An increasing body of literature has shown that grass endophytes provide numerous advantages to the host plant particularly in high-nutrient agronomic ecosystems [20, 21]. However, in the present study endophyte frequencies and vegetation productivity estimates were not linked (p<0.16). Totally endophyte-free populations were detected both in geographic sites with the lowest and highest productivity (Fig 5).

Although the study was not designed to test the importance of herbivore grazing, we acknowledge that our results support the previous studies suggesting that grazing by reindeer and sheep may promote high frequencies of endophyte infections in *F*. *rubra*. [15, 63]. Grasses of long term grazing-areas could be expected to have higher endophyte infection frequencies than that of low grazing areas due to endophyte-promoted grass resilience against herbivory [21, 64]. In our study all geographic sites with high endophyte incidence are heavily grazed whereas the totally endophyte-free populations (Hanko, Halti and Greenland) are moderately or rarely subjected to vertebrate grazers (Table 1). Grasslands in Faroe Islands and Iceland are heavily grazed by sheep, in Spain and Switzerland by large ungulates and in northern Finland by reindeer. However, grazing pressure may vary among populations. For example, reindeer density in Kevo area is twice as high (2.51–3.10 individuals/km^2^) as density in Halti area (1.01–1.50 individuals/km^2^) [65].

Our results show that both endophyte infection frequencies and polyploidization of red fescue are not associated with latitude and productivity as suggested by past studies [26, 46]. We propose that these results (1) mirror past distribution history of plants after glaciation period, and past or prevailing local selection forces such as herbivory grazing as suggested by geographic mosaic theory [66], and (2) productivity and latitude may only partly explain the frequencies of detected ploidy levels.

## Supporting Information

S1 Table Endophyte status and cytometric data of the plants with the geographic information, grazing level and mean normalized difference vegetation index (mean NDVI) of the collection sites. (XLS)

## References

[1] Wallace AR. The geographical distribution of animals New York: Harper and brothers; 1876.

[2] Stebbins GL. Chromosomal evolution in higher plants London: Addison-Wesley; 1971.

[3] Stebbins GL. Polyploidy and the distribution of the arctic-alpine flora: new evidence and a new approach. Botanica Helvetica. 1984;72: 824–832.

[4] Brochmann C, Brysting AK, Alsos IG, Borgen L, Grundt HH, Scheen AC, et al Polyploidy in arctic plants. Biological Journal of the Linnean Society. 2004;82: 521–536.

[5] Comai L. The advantages and disadvantages of being polyploid. Nature Reviews Genetics. 2005;6: 836–846. 10.1038/nrg1711 16304599

[6] Saikkonen K, Faeth SH, Helander M, Sullivan TJ. Fungal endophytes: A continuum of interactions with host plants. Annual Review of Ecology, Evolution and Systematics. 1998;29: 319–343.

[7] Clay K, Schardl CL. Evolutionary origins and ecological consequences of endophyte symbiosis with grasses. The American Naturalist. 2002;160: S99–S127. 10.1086/342161 18707456

[8] te Beest M, Roux JJL, Richardson DM, Brysting AK, Suda J, Kubesova M, et al The more the better? The role of polyploidy in facilitating plant invasions. Annals of Botany. 2012;109: 19–45. 10.1093/aob/mcr277 22040744 PMC3241594

[9] Kim S, Rayburn AL, Boe A, Lee DK. Neopolyploidy in *Spartina pectinata* Link: 1. Morphological analysis of tetraploid and hexaploid plants in a mixed natural population. Plant Systematics and Evolution. 2012;298: 1073–1083.

[10] Cheplick GP. Population biology of grasses Cambridge: Cambridge University Press; 1998.

[11] Aiken SG, Darbyshire SJ. Fescue grasses of Canada Ottawa: Canadian Government Publishing Centre; 1990.

[12] Sampoux JP, Huyghe C. Contribution of ploidy level variation and adaptive trait diversity to the environmental distribution of taxa in the 'fine-leaved' lineage (genus *Festuca* subg. *Festuca*). Journal of Biogeography. 2009;36: 1978–1993.

[13] Soltis DE, Soltis PS, Tate JA. Advances in the study of polyploidy since plant speciation. New Phytologist. 2003; 161: 173–191.

[14] Saikkonen K, Alholm J, Helander M, Lehtimäki S, Niemeläinen O. Endophytic fungi in wild and cultivated grasses in Finland. Ecography. 2000;23: 360–366.

[15] Granath G, Vicari M, Dawn R, Bazely DR, Ball JP, Puentes A, et al Variation in the abundance of fungal endophytes in fescue grasses along altitudinal and grazing gradients. Ecography. 2007;30: 422–430.

[16] Wäli PR, Ahlholm JU, Helander M, Saikkonen K. Occurrence and genetic structure of the systemic grass endophyte *Epichloe festucae* in fine fescue populations. Microbial Ecology. 2007;53: 20–29. 10.1007/s00248-006-9076-2 17186157

[17] Zabalgogeazcoa I, Gundel PE, Helander M, Saikkonen K. Non-systemic fungal endophytes in *Festuca rubra* plants. Fungal Diversity. 2013;60: 25–32.

[18] Clay K, Holah J. Fungal endophyte symbiosis and plant diversity in successional fields. Science. 1999;285: 1742–1744. 10481011 10.1126/science.285.5434.1742

[19] Schardl CL, Leuchtmann A, Spiering MJ. Symbioses of grasses with seedborne fungal endophytes. Annual Review of Plant Biology. 2004;55: 315–340. 10.1146/annurev.arplant.55.031903.141735 15377223

[20] Saikkonen K, Lehtonen P, Helander M, Koricheva J, Faeth SH. Model systems in ecology: dissecting the endophyte-grass literature. Trends in Plant Science. 2006;11:428–433. 10.1016/j.tplants.2006.07.001 16890473

[21] Saikkonen K, Saari S, Helander M. Defensive mutualism between plants and endophytic fungi? Fungal Diversity. 2010;41: 101–113.

[22] Adams KL, Wendel JF. Polyploidy and genome evolution in plants. Current Opinion in Plant Biology. 2005;8: 135–141. 10.1016/j.pbi.2005.01.001 15752992

[23] Ramsey J. Polyploidy and ecological adaptation in wild yarrow. Proceedings of the National Academy of Sciences. 2011;108: 7096–7101. 10.1073/pnas.1016631108 PMC3084070 21402904

[24] Yang X, Ye CY, Cheng ZM, Tschaplinski TJ, WullSchleger SD, Yin W, et al Genomic aspects of research involving polyploid plants. Plant Cell, Tissue and Organ Culture. 2011;104: 387–397.

[25] Pandit MK, White SM, Pocock MJ. The contrasting effects of genome size, chromosome number and ploidy level on plant invasiveness: a global analysis. The New Phytologist. 2014;203: 687–703. 10.1111/nph.12799 24697788

[26] Löve A, Löve D. Arctic polyploidy. Proceedings of the Genetics Society of Canada. 1957;2: 23–27.

[27] Těšitelová T, Jersakova J, Roy M, Kubatova B, Tesitel J, Urfus T, et al Ploidy-specific symbiotic interactions: divergence of mycorrhizal fungi between cytotypes of the *Gymnadenia conopsea* group (Orchidaceae). The New Phytologist. 2013;199:1022–1033. 10.1111/nph.12348 23731358

[28] Chao DY, Dilkes B, Luo HB, Douglas A, Yakubova E, Lahner B, et al Polyploids exhibit higher potassium uptake and salinity tolerance in *Arabidopsis*. Science. 2013;341: 658–659. 10.1126/science.1240561 23887874 PMC4018534

[29] Neiman M, Kay AD, Krist AC. Can resource costs of polyploidy provide an advantage to sex? Heredity. 2013;110: 152–159. 10.1038/hdy.2012.78 23188174 PMC3554456

[30] Gundel PE, Dirihan S, Helander M, Zabalgogeazcoa I, Väre H, Saikkonen K. Systemic fungal endophytes and ploidy level in *Festuca vivipara* populations in North European Islands. Plant Systematics and Evolution. 2014;300: 1683–1691.

[31] Afkhami ME, McIntyre PJ, Strauss SY. Mutualist-mediated effects on species' range limits across large geographic scales. Ecology Letters. 2014;17: 1265–1273. 10.1111/ele.12332 25052023

[32] Fine PEM. Vectors and vertical transmission: an epidemiologic perspective. Annals of the New York Academy of Sciences. 1975;266: 173–194. 829470 10.1111/j.1749-6632.1975.tb35099.x

[33] Ewald PW. Transmission modes and evolution of the parasitism–mutualism continuum. Annals of the New York Academy of Sciences. 1987;503: 295–306. 3304078 10.1111/j.1749-6632.1987.tb40616.x

[34] Kover PX, Clay K. Trade-off between virulence and vertical transmission and the maintenance of a virulent pathogen. The American Naturalist. 1998;152: 165–175. 10.1086/286159 18811383

[35] Lipstch MN. The population dynamics of vertically and horizontally transmitted parasites. Proceedings of the Royal Society London B. 1995;260: 321–327. 10.1098/rspb.1995.0099 7630898

[36] Herre EA, Knowlton N, Mueller UG, Rehner SA. The evolution of mutualism: exploring the paths between conflict and cooperation. Trends in Ecology & Evolution. 1999;14: 49–53. 10234251 10.1016/s0169-5347(98)01529-8

[37] Saikkonen K, Ion D, Gyllenberg M. The persistence of vertically transmitted fungi in grass metapopulations. Proceedings of the Royal Society London B. 2002;269: 1397–1403. 10.1098/rspb.2002.2006 PMC1691040 12079664

[38] Saikkonen K, Wäli P, Helander M, Faeth SH. Evolution of endophyte-plant symbioses. Trends in Plant Science. 2004; 9: 275–280. 10.1016/j.tplants.2004.04.005 15165558

[39] Saikkonen K, Ruokolainen K, Huitu O, Gundel PE, Piltti T, Hamilton CE, et al Fungal endophytes help prevent weed invasions. Agriculture, Ecosystems & Environment. 2013;165: 1–5.

[40] Bao G, Saikkonen K, Wang H, Zhou L, Chen S, Li G, et al Does endophyte symbiosis resist allelopathic effects of an invasive plant in degraded grassland? Fungal Ecology. 2015;17: 114–125.

[41] Song M, Li X, Saikkonen K, Li C, Nan Z. An asexual *Epichloë* endophyte enhances waterlogging tolerance of *Hordeum brevisubulatum*. Fungal Ecology. 2015;13: 44–52.

[42] Clay K. Fungal endophytes of grasses: A defensive mutualism between plants and fungi. Ecology. 1988;69: 10–16.

[43] Clay K. Fungal endophytes of grasses. Annual Review of Ecology, Evolution and Systematics. 1990;21: 275–297.

[44] Faeth SH. Are endophytic fungi defensive plant mutualists? Oikos. 2002;98: 25–36.

[45] Faeth SH, Helander ML, Saikkonen KT. Asexual *Neotyphodium* endophytes in native grass reduce competitive abilities. Ecoogy Letters. 2004;7: 304–313.

[46] Semmartin M, Omacini M, Gundel PE, Hernandez-Agramonte M. Broad scale variation of fungal-endophyte incidence in temperate grasses. Journal of Ecology 2015;103: 184–190.

[47] Paruelo JM, Epstein HE, Lauenroth WK., Burke IC. ANPP estimates from NDVI for the central grassland region of the United States. Ecology. 1997;78: 953–958.

[48] Saikkonen K, Taulavuori K, Hyvönen T, Gundel PE, Hamilton CE, Vänninen I, et al Climate change-driven species' range shifts filtered by photoperiodism. Nature Climate Change. 2012;2: 239–242.

[49] Dirihan S, Terho P, Helander M, Saikkonen K. Efficient analysis of ploidy levels in plant evolutionary ecology. Caryologia: International Journal of Cytology, Cytosystematics and Cytogenetics. 2013;66: 251–256.

[50] Darlington CD, La Cour F. The handling of chromosomes London: Allen & Unwin; 1969.

[51] Doležel J, Greilhuber J, Suda J. Estimation of nuclear DNA content in plants using flow cytometry. Nature Protocols. 2007;2: 2233–2244. 10.1038/nprot.2007.310 17853881

[52] Tucker CJ, Vanpraet CV, Sharman MJ, Ittersum GV. Satellite remote sensing of total herbaceous biomass production in the Senegalese Sahel: 1980–1984. Remote Sensing of Environment. 1985;17: 233–249.

[53] Prince S. Satellite remote sensing of primary production: comparison of results for Sahelian grasslands 1981–1988. International Journal of Remote Sensing. 1991;12: 187–216.

[54] Sellers PJ, Berry JA, Collatz GJ, Field CB, Hall FG. Canopy reflectance, photosynthesis, and transpiration. III. A reanalysis using improved leaf models and a new canopy integration scheme. Remote Sensing of Environment. 1992;42: 187–216.

[55] Huete AK, Didan K, Miura T, Rodriguez EP, Gao X, Ferreira LG. Overview of the radiometric and biophysical performance of the MODIS vegetation indices. Remote Sensing of Environment. 2002;83: 195–213.

[56] Piñeiro G, Oesterheld M, Paruelo J. Seasonal variation in aboveground production and radiation-use efficiency of temperate rangelands estimated through remote sensing. Ecosystems. 2006;9: 357–373.

[57] Nemani R, Running S. Land cover characterization using multitemporal red, near-IR, and ther mal-IR data from NOAA/AVHRR. Ecological Applications. 1997;7: 79–90.

[58] Zuur A, Ieno EN, Walker N, Saveliev AA, Smith GM. Mixed effects models and extensions in ecology with R New York: Springer Verlag; 2009.

[59] Bates D, Maechler M, Bolker B. lme4: Linear mixed-effects models using S4 classes. R package version 0.999375–39. 2011. Available: http://www.inside-r.org/packages/lme4/versions/0-999375-39.

[60] R Development Core Team. R: a language and environment for statistical computing R Foundation for Statistical Computing, Vienna 2011 Available: http://www.R-project.org/.

[61] Bartoń K. MuMIn: multi-model inference. R package, version 0.12.2. 2012 Available: http://r-forge.r-project.org/projects/mumin/.

[62] Harbed DJ. Observations on population structure and longevity of *Festuca rubra* L. The New Phytologist. 1961;60: 184–206.

[63] Bazel DR, Vicari M, Emmerich S, Filip L, Lin D, Inman A. Interactions between herbivores and endophyte-infected *Festuca rubra* from the Scottish islands of St. Kilda, Benecula Rum. Journal of Ecology. 1997;34: 847–860.

[64] Cheplick GP, Faeth S. Ecology and evolution of the grass endophyte symbiosis New York: Oxford University Press; 2009.

[65] Colpaert A, Kumpula J, Nieminen M. Reindeer pasture biomass assessment using satellite remote sensing. Arctic. 2003;56: 147–158.

[66] Thompson JN. The geographic mosaic of coevolution Chicago: University of Chicago Press; 2005.

